# Hepatoprotective and Anti-Oxidative Effects of Total Flavonoids From Qu Zhi Qiao (Fruit of Citrus Paradisi cv.Changshanhuyou) on Nonalcoholic Steatohepatitis *In Vivo* and *In Vitro* Through Nrf2-ARE Signaling Pathway

**DOI:** 10.3389/fphar.2020.00483

**Published:** 2020-04-22

**Authors:** Zheng Shi, Ting Li, Yuwen Liu, Tiantian Cai, Wendong Yao, Jianping Jiang, Yinghua He, Letian Shan

**Affiliations:** ^1^ Department of Pharmacy, The First Affiliated Hospital of Zhejiang Chinese Medical University (Zhejiang Provincial Hospital of Traditional Chinese Medicine), Hangzhou, China; ^2^ The First Affiliated Hospital, College of Medicine, Zhejiang University, Hangzhou, China; ^3^ Preparation Center, The First Affiliated Hospital of Zhejiang Chinese Medical University (Zhejiang Provincial Hospital of Traditional Chinese Medicine), Hangzhou, China; ^4^ Inspection Center of Traditional Chinese Medicine and Natural Medicine, Hangzhou Institute for Food and Drug Control, Hangzhou, China; ^5^ Research and Development Department, Zhejiang You-du Biotech Limited Company, Quzhou, China; ^6^ Institute of Orthopaedics and Traumatology, The First Affiliated Hospital of Zhejiang Chinese Medical University (Zhejiang Provincial Hospital of Traditional Chinese Medicine), Hangzhou, China

**Keywords:** total flavonoids from *Citrus*, nonalcoholic steatohepatitis, anti-oxidative, Nrf2, Nrf2-ARE signaling pathway

## Abstract

Nonalcoholic steatohepatitis (NASH) is a liver disease defined as the dynamic condition of hepatocellular injury during the progress of nonalcoholic fatty liver disease (NAFLD). Total flavonoids from the dry and immature fruits of *Citrus Paradisi* cv.*Changshanhuyou* (accepted species name: Citrus × aurantium L) (*Qu Zhi Qiao, QZQ*) are purified and named TFCH. This study was purposed to investigate and analyze the effect of TFCH on NASH model through Nuclear factor erythroid 2-related factor 2 (Nrf2)- antioxidant response elements pathway *in vivo* and *in vitro*. *In vivo* study was performed using male C57BL/6 mice fed with high fat diet 16 weeks for NASH model. After 7-week modeling, mice in TFCH-treated group were daily treated with intragastric administration of TFCH at 25 mg/kg, 50 mg/kg, 200 mg/kg, respectively, for successive 8 weeks. Histopathological and immunohistochemical analyses were conducted for evaluating severity of NASH-mice model and the effect of TFCH treatment. *In vitro* experiment was performed by using human LX-2 cells and cultured with Free fatty acid (FFA) (Oleic acid: palmitic: l: 0.5 mmol/L) for 24 h and then treated with TFCH at different concentrations (0, 25, 50, 100, 200 mg/ml) for 6 h,12 h, and 24 h. Anti-apoptosis effect of TFCH on LX-2 cells cultured with FFA was revealed by the CCK-8 assay. Lipid parameters and oxidative stress markers were measured *in vivo* and *in vitro*, results showed that TFCH dose-dependently and greatly increased the antioxidant ability and reduced the oxidative damage in NASH model. The protein expression of Nrf2 and the downstream target genes in mice liver and human LX-2 cells were tested by Western blot analysis to investigate the possible molecular mechanisms of TFCH. Our results indicated that TFCH up-regulated protein expression of these genes and have the significant influence in activating the Nrf2-ARE signaling pathway. This study shows Nrf2-ARE signaling pathway may provide novel therapeutic opportunities for NASH therapy in the future.

## Introduction

Nonalcoholic fatty liver disease (NAFLD) is the common liver disease wreaking havoc on people's lives. Prevalence of NAFLD in the world (only in adults) is surging to 24% at present(Younossi et al., 2018). Progress of NAFLD traces from simple steatosis to nonalcoholic steatohepatitis (NASH), and then pericellular fibrosis in NASH can slowly lead to cirrhosis and liver carcinoma. NASH patients developing fibrosis progression account for approximately 40%. Furthermore, the biggest immediate concern is the annual HCC incident rate in NASH patients was 5.29 per 1,000 person-years (Marjot et al., 2019). NASH is defined as the dynamic condition of hepatocellular injury, usually with ballooning as a hallmark, inflammation, and varying degrees of fibrosis, which have a highly stake in overweight and metabolic syndrome like the type 2 diabetes mellitus (Diehl and Day, 2017). Inflammation and oxidative stress are key factors in the pathogenesis of NASH, which can be applied as intervention targets for NASH treatment (Marra and Svegliati-Baroni, 2018). Elevated eactive oxygen species (ROS) generation causes damage in hepatocytes through systemic oxidative stress, triggering inflammation and fiborsis and ultimately leading to NASH (Spahis et al., 2017). Many studies demonstrated that Nrf2-ARE signaling pathway plays a key role in response to oxidative stress during NASH progress (Fisher et al., 2008; Lee et al., 2014). Therefore, oxidative stress plays a crucial role in pathogenesis of NASH and underscore solutions to rein and treat this disease is significant. The number of clinical trials and drug researches designed to identify effective and safe treatments for NASH are surging, but no agent has yet received approval by the Food and Drug Administration for the treatment of NASH (Cai et al., 2019). Drug therapy attached great importance to progressive NASH but also could increase risk of fibrosis progression in early-stage NASH. Guidelines for the management of NAFLD recommend improving obesity and diabetes, or bariatric (metabolic) surgery to reduce NASH progression (Carceller et al., 2016). Accordingly, there is an urgent need to develop new effective therapies.

Citrus species are natural products and notably rich in flavonoid compounds which are famous for multiple therapeutic benefits. Citrus flavonoids have been researching in recent years underlying its beneficial activities such as anti-oxidation, anti-inflammation, and anti-cardiovascular diseases (Bondonno et al., 2018; Vazhappilly et al., 2019). Additionally, some clinical trials found that plant flavonoids are effective in preventing obesity, type 2 diabetes, and some metabolic syndromes (Guo et al., 2016; Pan et al., 2017), which are strongly associated with NASH. Citrus species comprise a variety of fruits including sweet orange, mandarin, grape fruit, lemon, lime, and so on. Citrus fruits consist of an outer peel or rind (Ranganna et al., 1983), The cultivar *Citrus x paradisi* cv.*Changshanhuyou* (accepted species name: Citrus × aurantium L) are mainly produced in Changshan and Quzhou in Zhejiang Province, China. Fruits of *Citrus Paradisi* cv. *Changshanhuyou* are good source of flavonoids, a research showed that citrus peel extract could be used in obesity treatment (Nakajima et al., 2016). Total flavonoids from the dry and immature fruits of *Citrus Paradisi* cv.*Changshanhuyou* (accepted species name: Citrus × aurantium L) (*Qu Zhi Qiao, QZQ*), named TFCH and was reported in previously article (Jiang et al., 2019). TFCH, mainly composed of four flavonoids including narirutin, naringin, neohesperidin, and hesperidin, which is cheap and attainable in daily life. To date, only a few studies have dug into the potential implication of Citrus flavonoids in relation to possible anti-NASH benefits, and need to further investigation. Therefore, in order to identify the role of TFCH in NASH and elucidate the mechanisms of TFCH treatment, we purified TFCH from the peels of QZQ and established NASH model in animals and cells, with conducting molecular biological experiments.

## Materials and Methods

### Raw Material and Reagents

Fruits of *Citrus Paradisi* cv.*Changshanhuyou* (accepted species name: Citrus × aurantium L), which were collected from Changshan and Quzhou in Zhejiang Province, China. The fruits were cleaned, sliced, sundried, and purified, named *Qu Zhi Qiao* (*QZQ*). QZQ botanically identified by the authors (Voucher: JJ, 101011, ZM). The standardized material of QZQ (Qu Aurantii Fructus) was provided by Zhejiang Chinese Medical University Medical Pieces., Ltd., (Hangzhou, China) (batch number: 180916). The voucher specimen has been deposited at pharmacy department of the first affiliated hospital of Zhejiang Chinese Medical University.

All the reagents and material are of the analytical or highest grade. Low density lipoprotein cholesterol (LDL-C), High density lipoprotein cholesterol (HDL-C), Total cholesterol (TC) detection kit, Triglyceride (TG) detection kit, aspartate aminotransferase. detection kit, alanine aminotransferase detection kit, malondialdehyde (MDA) detection kit, Total Superoxide dismutase (SOD) detection kit, Free fatty acid (FFA) detection kit, and Glutathione Peroxidase (GSH-Px) detection kit were provided by Nanjing Jiancheng Biological Engineering Institute (Nanjing, China). 8-iso-PGF2α ELISA kit was obtained from MEIMIAIN (Wuhan, USA). CCK8 kit bought from Total protein extraction kit was bought from MedChemExpresss LLC (Shanghai, China). BCA protein quantification kit was bought from Solarbio (Beijing, China). RIPA and PMSF were the products of Beyotime (Shanghai, China). PVDF membrane was obtained from GE Healthcare Life Sciences (Beijing, China). Primary antibodies for nuclear factor-erythroid 2 related factor 2 (Nrf2), hemeoxygenase1 (*HO-1*), NAD(P)H: quinone oxidoreductase 1 (*NQO1*), r-glutamyl-cysteine synthetase (*r-GCS*), and Glutathione-S-Transferases (*GST*) were obtained from Affinity Biosciences (Shanghai, China). Secondary antibody is horseradish peroxidase labeled and purchased from Biological Reagents Company Limited (Shanghai, China).

Human stellate hepatocyte LX-2 cells were provided by Saibai Biotechnology Co., Ltd. (Shanghai, China). Dulbecco's modified Eagle's medium (DMEM), Trypsin 0.25% (1×) Solution, and phosphate buffer saline (PBS) were bought from Hyclone (Logan, UT, USA). Fetal bovine serum (FBS) purchased from Evergreen (Hangzhou, China). Protease inhibitors and phosphatase inhibitors were bought from CoWin Biosciences (Nanjing, China).

Standard material (≥99% of purity) of composition of QZQ were provided by the National Institute for the Control of Pharmaceutical and Biological Products (Beijing, China).

### Extraction and Purification of QZQ

Total flavonoids of QZQ (TFCH) were prepared as previously described (Jiang et al., 2019). Each 1,000 g peels of Fruits from QZQ was extracted with 0.10% Ca (OH)_2_ solution (with 3 times), then performed twice at 100°C for 1.5 h each time. Filtrates were mingled, decompressed, and concentrated total flavonoid concentration was 3.83 mg/ml. The total flavonoids were separated and enriched by HPD-300 macroporous resin. The amount of loaded samples and protocol to elute samples were same as previously described. The elution fraction was collected and enriched to dryness for use.

### Total Flavonoid Content of TFCH

Composition of TFCH was analyzed by High-performance liquid chromatography (HPLC) system, mainly including quaternary gradient pump, online degasser, UV detector, and column thermostat was from Shimadzu Corporation. Chromatographic separation was attained on a Hypersil SB C18 column (Thermo Fisher Scientific, MA, USA) at 25°C. The mobile phase consisted of water-acetonitrile and is eluted with a gradient method (elution gradient: 0–15 min, 20% acetonitrile; 15–35 min, 60%–100% acetonitrile; 42–45 min, 100%–20% acetonitrile; 45–50 min, 20% acetonitrile). The flow rate of mobile phase is 1.0 ml/min, wavelength was 283 nm, and samples injection volume was 10 μl. The flavonoids in the samples were depending on the chromatographic peaks of the standard substances of composition of TFCH (neohesperidin, naringin, narirutin, and hesperidin).

The content of total flavonoids of TFCH was referred to previous method. Briefly, each flavonoid extract was dissolved in methanol (1:1, w/v) and consecutively added with 0.5 ml of 10% Al (NO_3_)_3_ solution, handed with 0.5 ml of 5% NaNO2 solution. Then, the absorbance of the samples was measured at 0, 5, and 10 min (wavelength was 510 nm). The content of total flavonoids substance in TFCH was calculated using a standard curve.

### Animals and Modeling

A total of 48 healthy male SPF C57BL/6 mice weighed about 18g–22g, and were purchased from Shanghai Sippr-BK Laboratory Animal Co., Ltd.(production license: SCXK(Hu) 2013-0016) and housed in a controlled room (22 ± 2°C, 50%–60% relative humidity, 12 light/dark cycle) in Zhejiang Chinese Medical University Laboratory Animal Center (Grade SPF II, SCXK: 2013-0184) with free access to water and standard laboratory diet. Animal experimental protocols was approved by the Medical Norms and Ethics Committee of Zhejiang Chinese Medical University (Protocol number: ZSLL-2018-039). Mice experiments were performed according to the China legislation on the use and care of laboratory animals for minimizing animal suffering.

Mice were randomly divided into six groups of eight animals, NC to be negative control group, Model to be NASH model group, Vit E to be positive control group, TFCH-L as a group treated with low dose of TFCH (25 mg/kg), TFCH-M as a group treated with intermediate dose of TFCH (50 mg/kg), and TFCH-H group dieted with high dose of TFCH (100 mg/kg). Beside the negative control group was daily fed with normal feedstuff, other groups were daily fed with high-fat diet which consists of 82.5% of normal diet, 10% of lard, 5% of yolk powder, plus 2% of cholesterol, and 0.5% of cholate for 16 weeks for NASH modeling. After 7-week high-fat diet treating, mice in TFCH-treated group were daily fed with TFCH at 25 mg/kg, 50 mg/kg, 200 mg/kg with manner of intragastric administration respectively for successive 8 weeks. As the same time, mice in the Vit E group were orally treated with vitamin E at 100 mg/kg, and mice in the model and NC groups were treated with normal saline for 9 weeks. Randomly sacrificed three mice in the NC group and model group respectively at the end of 6 weeks. All other mice in each group were sacrificed under anesthesia following 16 weeks of treatment, and were fasted on water for 12 h the night before treatment. The liver and whole blood of mice were collected, as well as whole blood was centrifuged to obtain serum. A part of the liver was fixed with neutral formalin and embedded in paraffin for pathological observation. Another part of the liver was kept at -80°C meant to next step use.

### Biochemical Examination

The serum levels of ALT, AST, TG, TC, HDL-C, LDL-C were tested by an automatic biochemical analyzer. The serum levels of FFA, SOD, MDA, GSH-Px were tested by commercially available kits (Nanjing Jiancheng biological Engineering Institute). The 8-iso-PGF2α in serum was determined with a sandwich ELISA using a colorimetric commercial kit (MEIMIAN), in accordance with the manufacture instruction.

### Histological Analysis

At the end of the treatment, a part of the liver was fixed in 10% formalin 24 h at room temperature and paraffin embedded afterwards for histology and immunohistochemistry. Then, the paraffin-embedded sections were stained with hematoxylin-eosin and oil red O solution, to visualize the severity of steatohepatitis.

### Immunohistochemistry (IHC)

In brief, paraffin-embedded sections were dewaxed, rehydrated and treated for antigen retrieval, then incubated in 0.3% Triton X solution for 10 min and cleaned with PBS three times. After blocking with 5% BSA at room temperature for 30 min, the paraffin-embedded sections were incubated overnight at 4°C with 100 μl PBS-diluted primary antibodies against mice Nrf2. The sections were washed and incubated with secondary antibody at 37°C for 20 min and visualized using a 3,3′-diaminobenzidine (DAB) kit, then cell nuclei counterstained with hematoxylin. Sections were quantified in a blinded manner at 400× from six randomly selected fields.

### Enzyme-Linked Immunosorbent Assay (Elisa)

8-iso-PGF2α was directly quantified from mice serum. Mice serum were centrifuged at 3,500 r/min for 15 min, the serum was separated and subjected to 8-iso-PGF2α ELISA kits according to the manufacturer's instructions.

### Western Blot Analysis (WB)

The liver of mice was harvested by the lysate buffer with proteinase inhibitor and phosphatase inhibitor to obtain the whole liver protein lysate. Protein concentrations were determined using a BCA protein quantitative kit. Proteins were separated by denaturing sodium dodecyl sulfate polyacrylamide gel electrophoresis (SDS-PAGE) and transferred onto a PVDF membrane. The membrane was blocked with 5% defatted milk for 2 h, and then incubated overnight at 4˚C with the following primary antibodies: *Nrf2, HO-1, NQO1, r-GCS, GST*, GAPDH. After washed with TBST (5min) for three times, the membrane was incubated with secondary antibody for 2h at 4˚C and was visualized. The relative density of each band was applied to chemiluminescent indicator and was quantitatively detected.

### Cell Culture

Hepatic stellate cells (LX-2) were obtained from Saibai Biotechnology Co., Ltd (Shanghai, China) and cultured in DMEM supplied with 10% FBS at 37°C under humidified atmosphere of 5% CO2.

### Cell Viability Assay (CCK-8 Assay)

LX-2 cells were seeded on 96-well plates, besides the negative control group, LX-2 cells in other groups were incubated in medium containing with FFA (Oleic acid: palmitic: l: 0.5 mmol/L) for 24 h, and then cultured with TFCH at different concentrations (0, 25, 50, 100, 200 mg/ml) for 6 h, 12 h, and 24 h. 10 ul Cell Counting Kit-8 solution was added to each well and then the plates were incubated for 2 h at 37°C to measure LX-2 cells viability.

### Cell Biochemical Examination and Signaling Pathways

LX-2 cells were incubated in medium containing with FFA and treated with TFCH at 100 ug/L for 12 h, then the biochemical level of AST, LDH, TG, MDA, SOD, GSH-Px in LX-2 cells were tested by an automatic biochemical analyzer and commercially available kits.

### Western Blot Analysis

The LX-2 cells (1.5x10^6^) were harvested by using RIPA lysis buffer with proteinase inhibitor cocktail to collect total protein lysate. Proteins extractions were separated by SDS-PAGE and transferred to nitrocellulose membranes. The membrane was blocked with 5% defatted milk for 2h and incubated overnight at 4˚C with the following primary antibodies: Nrf2*, HO-1, NQO1, r-GCS, GST*, GAPDH. After the membrane was washed, the relative density of each band was applied to chemiluminescent indicator and was quantitatively detected.

### Statistical Analysis

Data were expressed as the mean ± standard deviation (SD), and a *P*-value < 0.05 was considered to indicate a significant difference and a *p*-value < 0.01 considered to indicate a very significant difference. Comparison between groups used T Test (homogeneity of variance), Kruskal-Wallis H Test (heterogeneous variance) and using one-way ANOVA followed by Fisher's least significant difference (LSD) comparison.

## Results

### Total Flavonoid Content of TFCH

**Figure 1:**
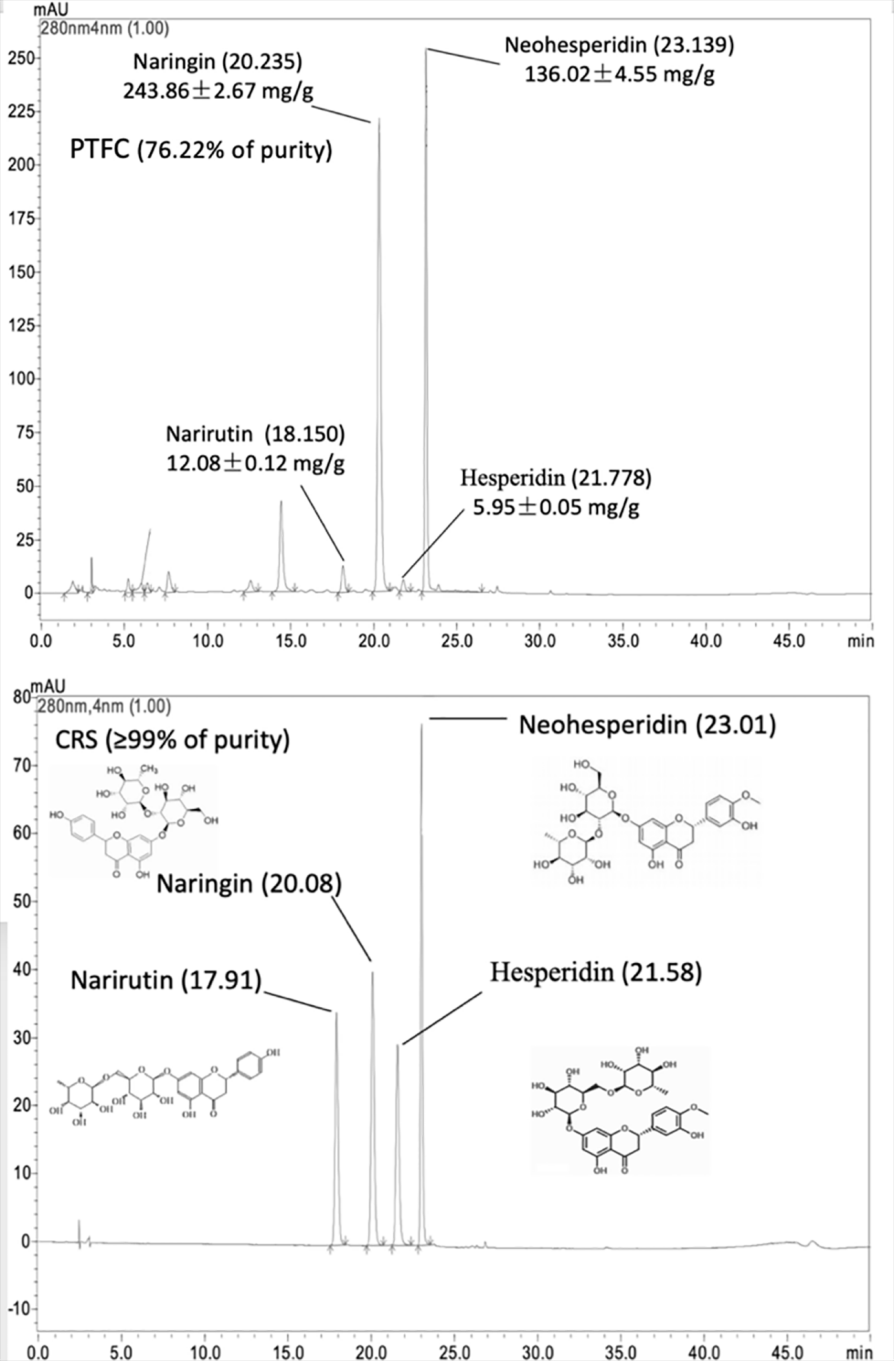
High-performance liquid chromatography (HPLC) chromatographic profile of TFCH and standard substances of narirutin, naringin, neohesperidin, and hesperidin.

The HPLC chromate-graphic profile of TFCH is shown in **Figure 1**. The HPLC chromate-graphic profile indicated that TFCH contained narirutin, naringin, neohesperidin and hesperidin respectively, as well as the peaks of retention times were at 17.910 min, 20.077 min, 21.567 min, and 23.011 min. Through using rutin equivalents, the total flavonoid content (purity) of TFCH was 76.22%. The content of narirutin, naringin, neohesperidin, and hesperidin in TFCH were calculated according to the standard curve, which contained 12.08± 0.12 mg/g, 243.86 ± 2.67 mg/g, and 136.02 ± 4.55 mg/g, respectively.

### *In Vivo* Anti-NASH Effect of TFCH on Biomarkers and Oxidative Stress Markers

**Figure 2:**
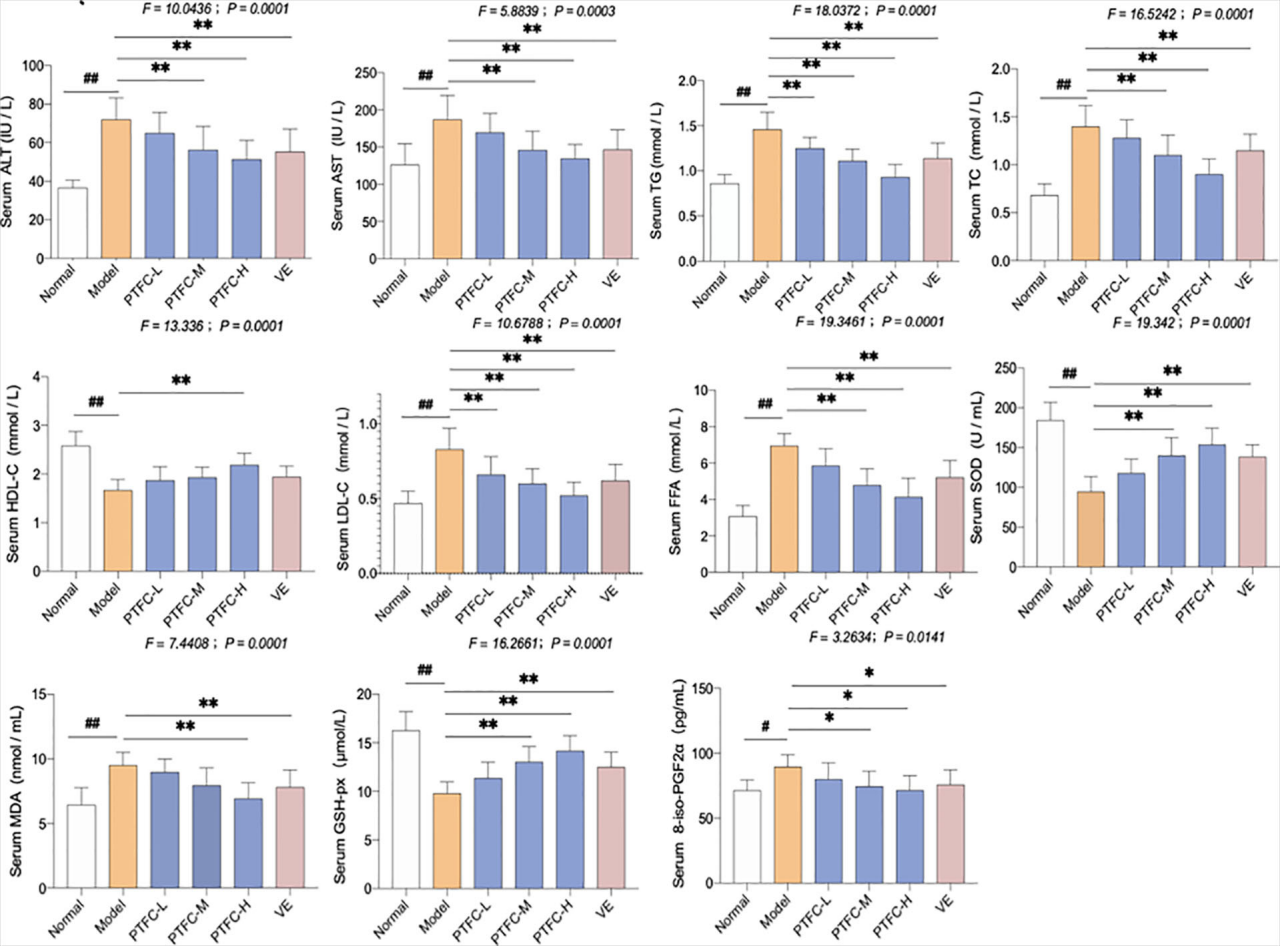
Levels of biochemical parameters and oxidative stress markers in the serum and livers of mice. Values are shown as mean ± SD (*n* = 8). Data (mean ± SD) are statistically different with each other at LSD multiple comparisons, ***^#^****p* < 0.05 or ***^##^****p* < 0.01 vs. NC group; *p < 0.05 or **p < 0.01 vs. model group.

The lipid profiles in mice serum and livers are shown in **Figure 2**. High-fat diet feeding markedly enhanced serum levels of ALT, AST, FFA, TG, TC, and LDL-C in mice, while decreased HDL-C level in mice (all *p* < 0.01 vs. NC levels). However, treatment of TFCH decreased serum levels of lipid parameters (ALT, AST, TG, FFA, TC, LDL-C) and increased HDL-C level in NASH mice fed with high-fat diet. Hence, TFCH was capable of alleviating NASH-induced hyperlipidemia in a dose-dependent manner (all *p* < 0.05 or *p* < 0.01 vs. model levels excepted TFCH-L group). Although the significant difference of serum HDL-C was only observed between the TFCH-L group and model group.

The oxidative stress markers including SOD, GSH-Px, MDA, and 8-iso-PGF2α activity in serum were also measured. In model group, SOD and GSH-Px levels decreased, while MDA and 8-iso-PGF2α levels significantly increased (all *p* < 0.05 or *p* < 0.01 vs. NC levels). However, treatment with TFCH increased the SOD and GSH-Px while decreased the MDA and 8-iso-PGF2α expression levels in serum of high-fat diet treated mice (all *p* < 0.05 or *p* < 0.01 vs. model levels excepted TFCH-L group). However, the significant difference of MDA was only observed between the TFCH-L group, VE group, and model group. TFCH reversed these parameter changes in a dose-dependent manner, and the effects of TFCH-H and VE were similar.

### Anti-NASH Effect of TFCH on Histological Changes and Immunohistochemistry

**Figure 3:**
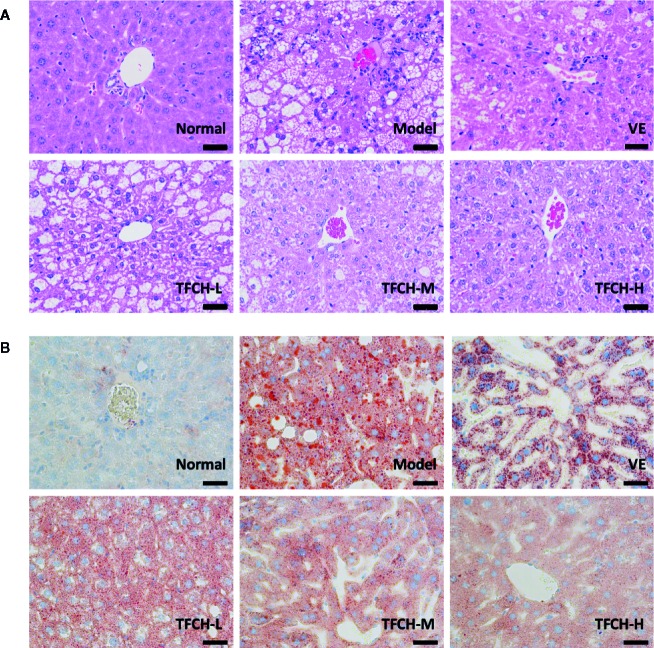
**(A)** Histopathological observation of HE staining on livers of mice. Images were obtained at 400× magnification (scale bar=50μm). **(B)** Histopathological observation of oil red O staining on livers of mice. Images were obtained at 400× magnification (scale bar=50μm).

Histopathological results of HE staining and oil red O staining were shown in **Figures 3A, B**, respectively. As seen in HE staining, the hepatic lobule structure was regular and without fat accumulation in hepatocytes or accumulation of inflammatory cells in the NC group. In model group, the liver sections showed apparent inflammatory damage and had hepatic steatosis with significant changes, including swelling and necrosis of hepatocytes, steatosis, and portal inflammatory cell infiltration. Degenerate phenotype is gradually reversed in a dose-dependent manner by TFCH treatment, the livers treated with high dose of TFCH (TFCH-H) showed obvious improvement including attenuating hepatic steatosis and hepatic lipogenesis, which seemed similar to that treated with VE.

In oil red O staining, there were no lipid observed in the liver tissue of the NC group and the cell morphology was normal. compared with the NC group, a large number of lipid droplets were distributed in the liver tissue of the model group, besides cells in model group became round and the cells were not tightly bound. The number and distribution of lipid droplets were significantly reduced by TFCH treatments in different dose manner. The degree of lipid droplet agglomeration: model group > TFCH-L group > TFCH -M group > TFCH -H group > VE group > NC group.

**Figure 4:**
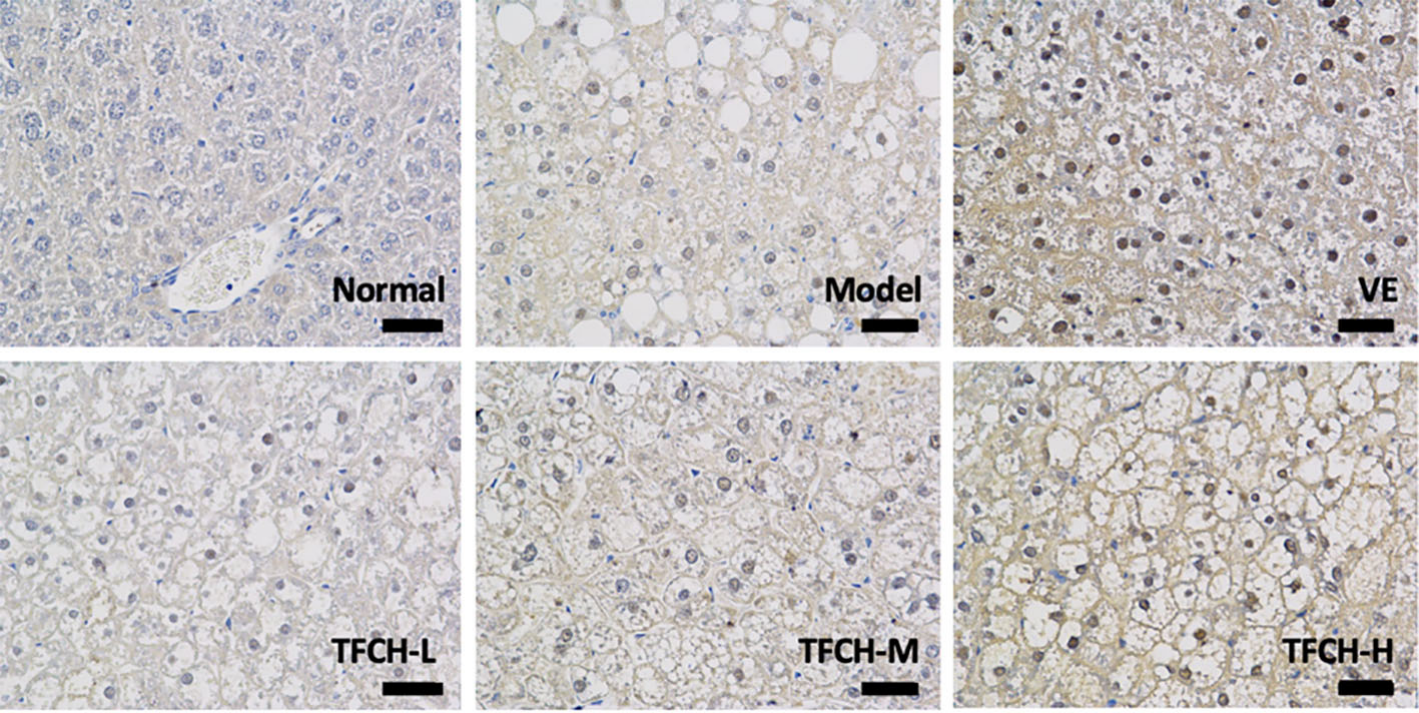
Immunohistochemistry observation of nuclear factor erythroid 2-related factor 2 (Nrf2) expressions in livers of mice. Images were obtained at 400× magnification (scale bar=50μm).

Results of immunohistochemical analysis were shown in **Figure 4**. The NC group showed normal expression of Nrf2 in liver. Compared with model group, the Nrf2 expression was obviously upregulated and its positive area significantly increased by TFCH in a dose-dependent manner, and it even reached a VE-like phenotype with TFCH-H treatment.

### Nrf2 Signaling Pathway Related Mechanism of TFCH in Liver

**Figure 5:**
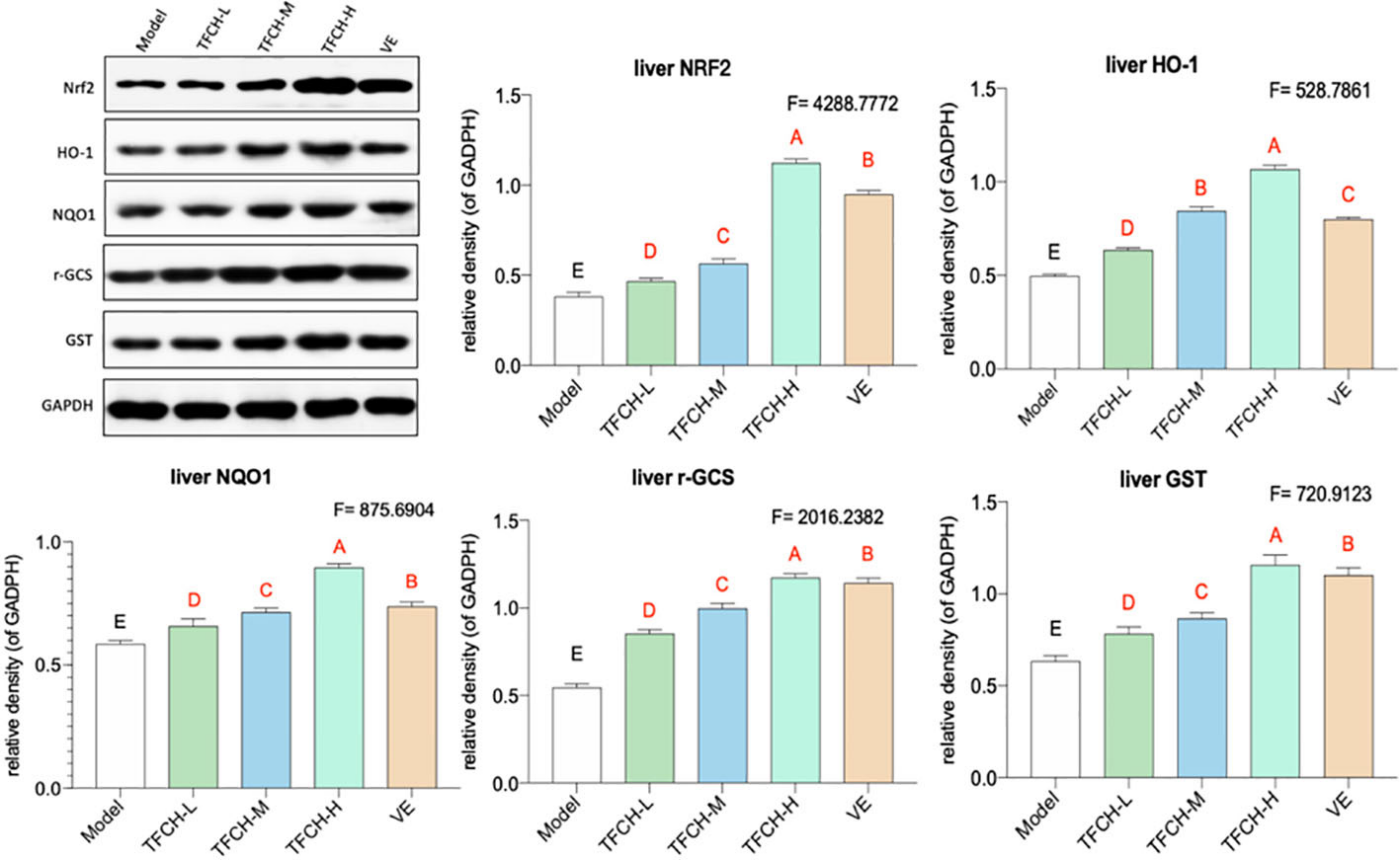
Expression of the targeted proteins in mice liver. Values are shown as mean ± SD (*n* = 8). Data (mean ± SD) with different case letters are statistically different with each other at LSD multiple comparisons, which with red letters differed significantly as compared to model group level. ^uppercase letter^ *p* < 0.01 vs. another group.

As shown in **Figure 5**, the influence of TFCH on the expression levels of targeted proteins in the liver of mice were measured by WB. Compared with NASH model group, the liver protein expression of Nrf2 and the Nrf2-controlled anti-oxidative genes *HO-1*, *NQO1*, *r-GCS*, and *GST* were significantly up-regulated by TFCH treatments and concentration-dependent increase in Nrf2 and the Nrf2-controlled antioxidant genes levels were also observed (all *p <*0.01vs model group). In addition, TFCH at higher dose have better regulation than VE. This suggests that anti-oxidative effect of TFCH on the NASH through the Nrf2 signaling pathway.

### Anti-NASH Effect of TFCH in LX-2 Cells

**Figure 6:**
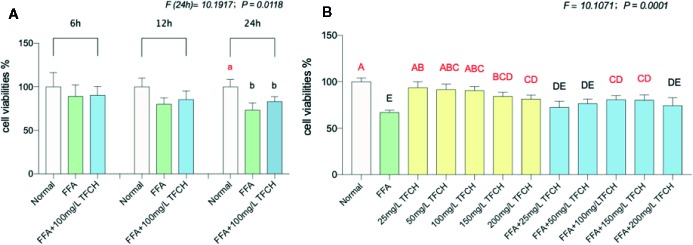
Cell viabilities of LX-2 cells were assayed by CCK-8. **(A)** Cell viability of LX-2 cells in each group (normal group; free fatty acid [FFA] group; FFA-100 mg/L TFCH group) were measured at 6 h, 12 h, and 24 h. **(B)** Cell viability of FFA-induced LX-2 cells in different TFCH group were measured at 24 h. Values are shown as mean ± SD (n =3). Data (mean ± SD) with different case letters are statistically different with each other at LSD multiple comparisons, which with red letters differed significantly as compared to model group level; ^uppercase lette^ *^r^ p <*0.01 vs. another group; ^lowercase letter^ *p* < 0.05 vs. another group.

The cell proliferation inhibitory effect of TFCH was determined by CCK-8 assay. As shown in **Figure 6**, TFCH significantly promoted the cell viability of LX-2 cells in a dose-dependent manner ranging from 25 to 200 μg/ml. We employed TGCG in 100 μg/ml doses with FFA at 6 h, 12 h, 24 h treatments for the next assessments. Compared with FFA model group, the proliferative effects on LX-2 cells, TFCH showed a stronger effect at 24 h treatment (**Figure 6A**) (*p <*0.05 vs NC group), although there is no significant difference of cell viabilities was observed between the TFCH treated group and model group. The treatment behavior of TFCH was in no time-dependent manner.

As shown in **Figure 6B**, TFCH derived from a dose range of 25 mg/L–200 mg/L significantly promote proliferation of the FFA-induced LX-2 cells at a dose-dependent manner with survival rate from 80.86 ± 4.34% to 72.66 ± 6.4% at 24 h. Moreover, there showed significant difference at 100 μg/ml and 150 μg/ml dose of TFCH versus the FFA model group. Thus, the proliferative effect of TFCH on FFA-induced LX-2 cells ranging from 25 to 100 μg/ml was in dose-dependent manner and has the best effect at 100 μg/ml dose.

### *In Vitro* Anti-NASH Effect of TFCH on Biomarkers and Oxidative Stress Markers

**Figure 7:**
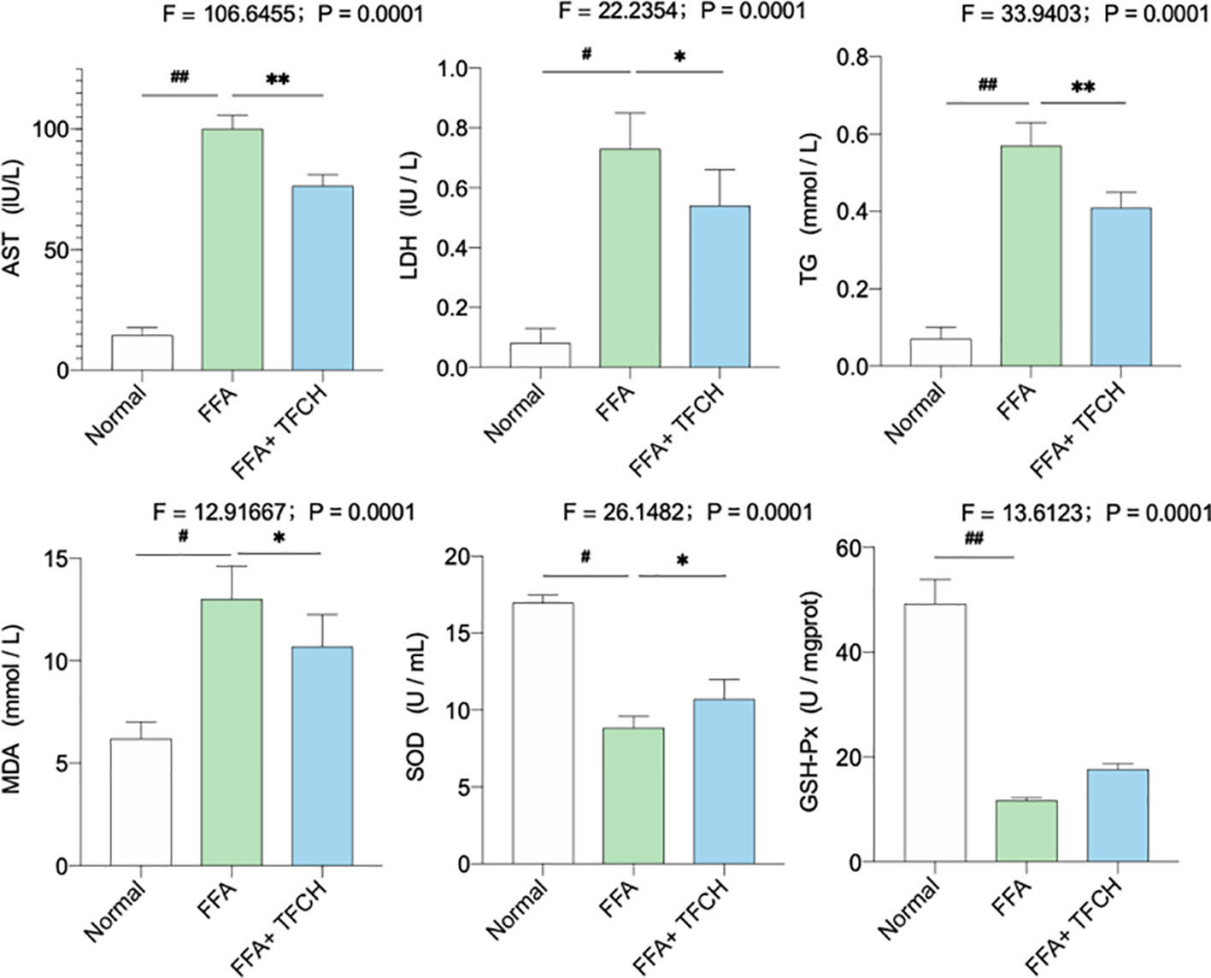
Levels of biochemical parameters and oxidative stress markers in the LX-2 cells. Values are shown as mean ± SD (n =3). Data (mean ± SD) are statistically different with each other at LSD multiple comparisons, **^#^***p* < 0.05 or **^##^***p* < 0.01 vs. NC group; *p < 0.05 or **p < 0.01 vs. model group.

As illustrated in **Figure 7**, significant alterations in serum lipid parameters (AST, LDH, TG, MDA, SOD, and GSH-PX) induced by FFA-cultured in the NAFLD model group (all *p <*0.05 or *p* < 0.01 vs. NC levels). Treatments of TFCH decreased the levels of AST, LDH, TG, MDA, as well as increased SOD and GSH-PX level in LX-2 cells cultured with FFA (all *p <*0.0*5* or *p <*0.01 vs model group). However, there is no significant difference of GSH-PX was shown between the TFCH group and model group. The alterations of those parameters were significantly influenced by TFCH group and showed the protective effect of TFCH on LX-2 cells cultured with FFA.

### The Nrf2-ARE Signaling Pathway Related Anti-Oxidative Mechanism of TFCH on LX-2 Cells

**Figure 8:**
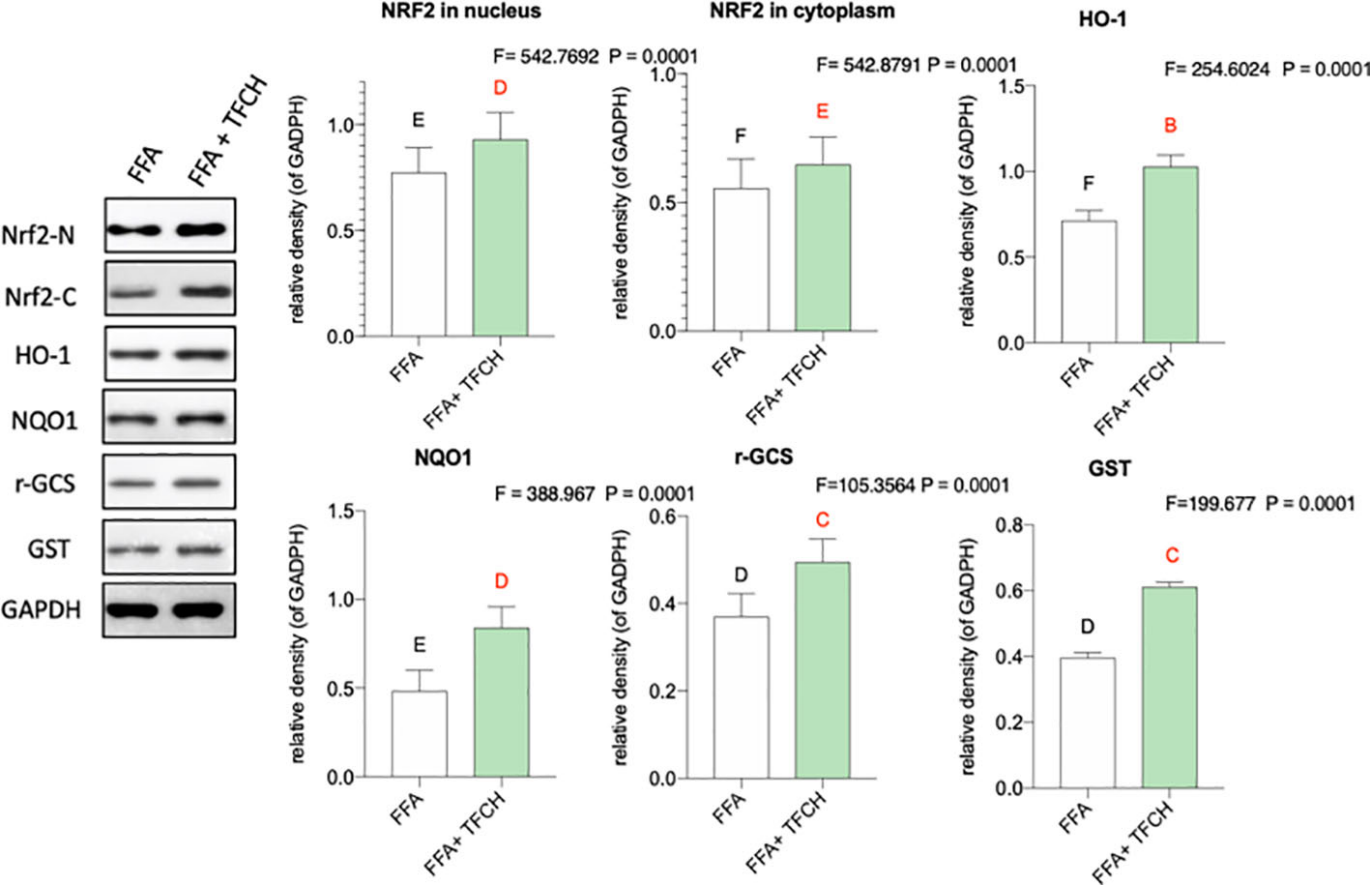
Effect of TFCH on Nrf2 and the downstream target genes in the LX-2 cells cultured with free fatty acid (FFA). Values are shown as mean ± SD (*n* =3). Data (mean ± SD) with different case letters are statistically different with each other at LSD multiple comparisons, which with red letters differed significantly as compared to model group level. ^uppercase letter^ *p* < 0.01 vs. another group.

As present in **Figure 8**, compared with FFA-induced model group, Nrf2 and the Nrf2-controlled antioxidant genes (*HO-1*, *NQO1*, *r-GCS*, and *GST*) *in vitro* were upregulated by TFCH at protein levels. While no significant association was found between hepatic protein levels of Nrf2 and any other Nrf2-mediated antioxidant proteins. As a result, this increase seems to positively correlate with TFCH treatments, indicating the TFCH showed a cellular protection response to oxidative stress by regulation of the Nrf2 signaling pathways (all *p* < 0.01 vs model group).

## Discussion

**Figure 9:**
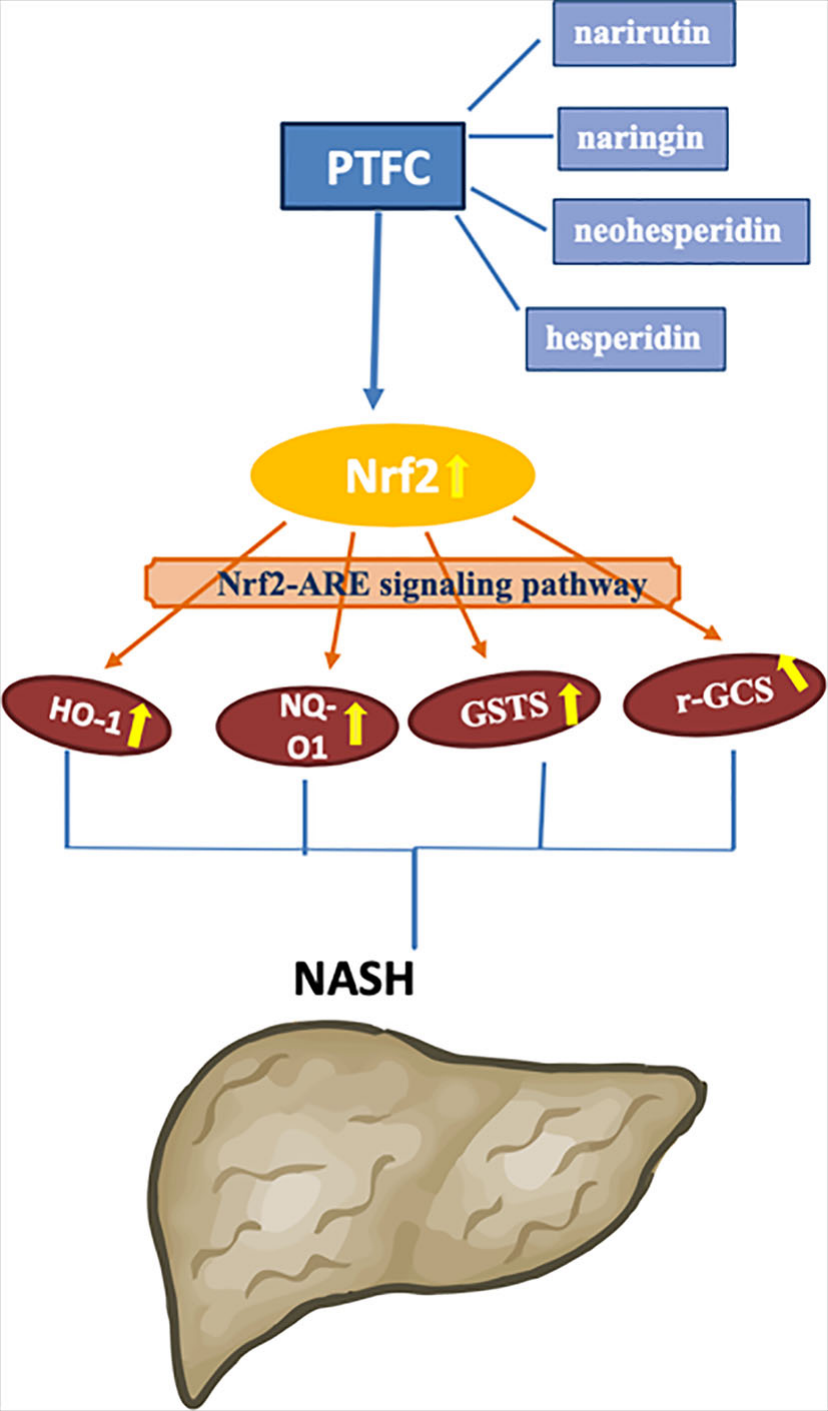
Proposed mechanism of TFCH against nonalcoholic steatohepatitis (NASH).

As a progressive form of NAFLD, NASH tendentiously develops to cirrhosis and hepatocellular carcinoma (Pittala et al., 2019). Inflammation and oxidative stress are key factors in the pathogenesis of NASH, which can be applied as intervention targets for NASH treatment (Marra and Svegliati-Baroni, 2018). Our previous study has demonstrated anti-inflammatory effect of TFCH against NAFLD, indicating potential anti-NASH efficacy of this natural product (Jiang et al., 2019). In this study, we evaluated the anti-NASH efficacy of QZQ-derived TFCH by using high-diet mice and cell model, revealing hepatoprotective and anti-oxidative effects of TFCH. The mechanism of TFCH was mediated by the Nrf-ARE signaling pathway (**Figure 9**). This is the first report on the anti-NASH efficacy and the mechanism of TFCH, which provides novel evidence of natural products for NASH treatment.

Lipid peroxidation-induced lipotoxicity and hepatic damage is the initial cause of NASH, in which increasing amounts of free fatty acids and cholesterols present to affect the behavior and function of hepatocyte (Marra and Svegliati-Baroni, 2018). Therefore, the levels of free fatty acids and cholesterols, such as HDL-C, LDL-C, TC, TG, and FFA, are used as biomarkers of lipotoxicity (Galluzzi et al., 2012). When hepatocytes are damaged, transaminases, such as ALT and AST, will be released from liver to blood, indicating serum ALT and AST as biomarkers of liver damage (Shi et al., 2017). In this study, these parameters were abnormal in the NASH model group and were reversed by TFCH, indicating a hepatoprotective effect of TFCH (**Figures 2** and **7**). SOD and GSH-Px are the primary antioxidant enzymes for liver protection against oxidative stress, which eliminates lipid peroxides and ROS from liver (Ferreira et al., 2005; Bao et al., 2010). On the contrary, MDA and 8-iso-PGF2α are final products of lipid peroxidation and can indirectly reflect the degree of liver damage caused by free radicals (Da Silva et al., 2017). In our study, the antioxidant markers were from serum samples. Though the markers from serum samples may not precisely reflect the capabilities of liver, increased serum antioxidant markers are consequent to the liberation of ROS in liver tissue (El-Shabrawi et al., 2014). With TFCH treatment, the activities of SOD and GSH-Px were increased, while that of MDA and 8-iso-PGF2α were decreased, indicating an anti-oxidative effect of TFCH (**Figures 2** and **7**).

As a chronic metabolic liver disease, NASH contains simple fatty liver, fatty liver fibrosis, and associated cirrhosis, induced by oxidative stress and inflammation (Byrne and Targher, 2015; Liu et al., 2019). The pathogenesis of NASH is closely associated with Nrf2-ARE signaling pathway, since Nrf2-ARE plays a key role in response to oxidative stress during NASH progress (Fisher et al., 2008; Lee et al., 2014). Up-regulation of Nrf2-ARE signaling activity is important in reversing many oxidative-stressed diseases, such as NASH (Sampath et al., 2019; Tao et al., 2019). Recently, it becomes a new target of treatment in liver injury and liver fibrosis (Shin et al., 2013). Nrf2 is an oxidative stress-mediated transcription factor regulating a variety of genes, such as *HO-1*, *GSTS*, *NQO1*, and *r-GCS*, by binding to ARE (Itoh et al., 2010). These genes function to resist oxidative damage on liver. For example, *HO-1* expresses with reactive oxygen species (ROS) generation to protect cells by increasing antioxidant products biliverdin, bilirubin, and CO (Loboda et al., 2016). *GST* encodes a detoxifying enzyme which can suppress ROS activation (Cavin et al., 2008). *NQO1* is a gene of cytoprotective antioxidant for defensing the ROS-induced cell damage (Nguyen et al., 2009; Zhang et al., 2017). *r-GCS* encodes a catalyzer for the synthesis of Glutathione which acts as an intracellular antioxidant (Gould and Pazdro, 2019). Thus, up-regulation of Nrf2 and its target genes could decrease ROS generation and keep oxidant/antioxidant balance. ROS is the by-products of normal metabolism that produced in liver cells. Elevated ROS generation causes damage in hepatocytes through systemic oxidative stress, triggering inflammation and fiborsis and ultimately leading to NASH (Spahis et al., 2017). Therefore, Nrf2-ARE signaling pathway is important for NASH development and also for its treatment. In this study, as shown in **Figures 5** and **8**, compared with model group, Nrf2 and its target genes (*HO-1, NQO1, GST*, and *r-GCS*) were up-regulated by TFCH treatments *in vivo* and *in vitro*, demonstrating the therapeutic mechanism of TFCH against NASH was related with Nrf-ARE signaling.

Flavonoids product has numerous biological properties, which may be promising therapeutic agents for NASH (Li and Schluesener, 2017). Many natural products are rich in flavonoids and possess anti-oxidative and anti-inflammatory activities against NASH (Jian et al., 2018; Parafati et al., 2018; Wang et al., 2018). Various flavonoid compounds have been identified, such as flavones, flavanones, flavanols, flavonols, and chalcones (Rzepecka-Stojko et al., 2015). In this study, TFCH contains narirutin, naringin, neohesperidin, and hesperidin, with 76.22% purity of total flavonoid content. The four flavonoids are the major active components in *Citrus* species, which exert strong anti-oxidant activity to scavenge superoxide radicals, prevent lipid peroxidation, and restore the antioxidant enzymes (e.g., SOD, GST, and GSH-Px) in liver. (Cavia-Saiz et al., 2010; Pari and Amudha, 2011; Sun et al., 2013; Sun et al., 2019). The anti-oxidant activity of these compounds was related to the regulation of Nrf signaling molecules, such as *HO-1* and *GST* (Suarez et al., 1998; Parhiz et al., 2015). Therefore, these compounds might play key roles in the hepatoprotective and anti-oxidant effects and mechanism of TFCH. In future, the anti-NASH effect and mechanism of each compound.

The preparation process of TFCH has been developed as a new Chinese Invention Patent (ZL 201110269192.2) by us, since high-purified flavonoid content has been obtained in our product. Although four flavonoid compounds in TFCH have been identified, their specific functions and contributions to the anti-NASH efficacy of TFCH remain unclear and require further investigation. These different flavones of TFCH for NASH should be further explored. The flavonoid purity of TFCH attained 76.22%, which was higher than that of other like products. Nevertheless, the chemical composition and bioactivity of the residual part (23.78%) are unknown yet and should be concerned in future studies.

## Conclusion

TFCH are the flavonoids purified from *Qu Zhi Qiao* (*QZQ*) which belong to the dry and immature fruits of *Citrus Paradisi* cv.*Changshanhuyou* (accepted species name: Citrus × aurantium L). TFCH contains narirutin, naringin, neohesperidin, and hesperidin, respectively. We present evidence that TFCH has hepatoprotective and anti-oxidative effect on NASH *in vivo* and *in vitro* mediated by regulating Nrf2-ARE signaling pathway. TFCH treatment may provide a novel therapeutic opportunity for NASH therapy in the future.

## Data Availability Statement

The raw data supporting the conclusions of this article will be made available by the authors, without undue reservation, to any qualified researcher.

## Ethics Statement

The animal study was reviewed and approved by The Institutional Animal Care and Use Committee of Zhejiang Chinese Medical University.

## Author Contributions

ZS and TL performed the main experiments. YL contributed to the TFCH preparation and quality control. JJ designed the experiments, wrote the manuscript, and revised the manuscript. TC and WY improved the experimental design and methodology. YH contributed to the revision. LS improved the design, draft, revision, and language editing of this manuscript. All authors agreed the submission of this manuscript and agreed to be accountable for all aspects of this work.

## Conflict of Interest

Corresponding author JJ was employed by the Zhejiang You-du Biotech Limited Company.

The remaining authors declare that the research was conducted in the absence of any commercial or financial relationships that could be construed as a potential conflict of interest.

## Abbreviations

ALT, alanine aminotransferase; AST, aspartate aminotransferase; FBS, Fetal bovine serum; FDA, Food and Drug Administration; FFA, Free fatty acid; GST, Glutathione-S-Transferases; HCC, hepatocellular carcinoma; HDL-C, High density lipoprotein cholesterol; HO-1, hemeoxygenase1; HPLC, high-performance liquid chromatography; H&E, hematoxylin and eosin; GSH-Px, Glutathione Peroxidase; LDL-C, low density lipoprotein cholesterol; LSD, least significant difference; MDA, malondialdehyde; NAFLD, Nonalcoholic fatty liver disease; NASH, Nonalcoholic steatohepatitis; Nrf2, factor-erythroid 2 related factor 2; NQO1, NAD(P)H: quinone oxidoreductase 1; PBS, Solution and phosphate buffer saline; QZQ, Qu Zhi Qiao; r-GCS, r-glutamyl-cysteine synthetase; SD, standard deviation; SDS-PAGE, sodium dodecyl sulfate polyacrylamide gel electrophoresis; SOD, Total Superoxide dismutase; TC, total cholesterol; TFCH, total flavonoids from *Citrus Paradisi* cv.*Changshanhuyou*; TG, Triglyceride; WB, Western blot.
